# Nephrotic syndrome associated with ramucirumab therapy: A single-center case series and literature review

**DOI:** 10.1097/MD.0000000000016236

**Published:** 2019-07-05

**Authors:** Teruhiro Fujii, Kentaro Kawasoe, Akiko Tonooka, Akihito Ohta, Kosaku Nitta

**Affiliations:** a Division of Nephrology, Department of Medicine, Department of Medicine, Tokyo Metropolitan Komagome Hospital; b Department IV, Internal Medicine, Tokyo Women's Medical University; c Department of Pathology, Department of Medicine, Tokyo Metropolitan Komagome Hospital, Japan.

**Keywords:** nephrotic syndrome, proteinuria, ramucirumab, vascular endothelial growth factor

## Abstract

Ramucirumab is a human immunoglobulin G1 monoclonal antibody that binds to vascular endothelial growth factor receptor 2 and is used for the treatment of metastatic or inoperable gastric, colorectal, and non-small cell lung cancers. However, ramucirumab can result in renal adverse events, including nephrotic syndrome, and the clinical course of this event is unclear. This study aimed to investigate the clinical course and pathological findings of patients with nephrotic syndrome after ramucirumab treatment.

We evaluated 5 patients with malignancies (2 cases of gastric cancer and 3 cases of colorectal cancer) who developed nephrotic syndrome during treatment with ramucirumab. Two patients were diagnosed based on renal biopsy. We investigated the relationship between ramucirumab treatment and clinical courses, pathological findings, and renal outcomes.

Four of 5 patients developed nephrotic syndrome after 1 or 2 doses of ramucirumab. All patients had hypertension, and 2 of 5 patients had renal dysfunction, defined as an increase in serum creatinine levels of ≥50% or ≥0.3 mg/dL. The 2 renal biopsy samples revealed a diffuse glomerular basement membrane double contour, intracapillary foam cell infiltration, and partial foot process effacement. Early drug discontinuation and antihypertensive therapy improved proteinuria, renal dysfunction, and hypertension in all patients.

Nephrotic syndrome is a renal adverse event observed in cancer patients after ramucirumab treatment. We suggest that urinalysis, renal function, and blood pressure should be closely monitored in patients undergoing ramucirumab treatment, and treatment should be discontinued if renal adverse events are detected.

## 1 Introduction

Anti-vascular endothelial growth factor (VEGF) therapies are efficacious in treating solid tumors.^[1,2]^ Anti-VEGF therapies include neutralizing anti-VEGF antibodies and treatments targeting the VEGF receptor (VEGFR). Ramucirumab is a human immunoglobulin G1 monoclonal antibody that binds to VEGFR-2 and is used to treat metastatic and/or inoperable gastric, colorectal, and non-small cell lung cancer. The RAINBOW study demonstrated that ramucirumab plus paclitaxel lengthened progression-free survival (PFS) compared with placebo plus paclitaxel; overall survival (OS) was also longer in the ramucirumab group than in the placebo group. In addition, the RAISE study on colorectal cancer and the REVEL study on non-small cell carcinoma also demonstrated that PFS and OS improved with ramucirumab as second-line therapy.^[3–5]^

Common toxicities associated with ramucirumab include bleeding, diarrhea, fatigue, nausea, and laboratory abnormalities, such as neutropenia, thrombocytopenia, and anemia. The reported renal adverse effects of ramucirumab mainly consist of proteinuria. Meta-analysis revealed that ramucirumab administration was associated with incidence rates of 9.4% for all grades of proteinuria, 1.1% for grade ≥3 proteinuria, and 0.1% for nephrotic syndrome.^[6]^

Furthermore, many cases of nephrotic syndrome have been induced by anti-VEGF therapies.^[7]^ However, few cases of nephrotic syndrome with ramucirumab treatment have been reported.^[8,9]^ To better understand the course of such patients, we investigated the clinical courses and pathological findings of nephrotic syndrome in 5 patients treated with ramucirumab.

## 2 Methods

### 2.1 Patients and definitions

This case series was approved by the institutional review committee of the Komagome Hospital (approval certificate number 2256) and was carried out according to the principles outlined by the Declaration of Helsinki. We collected clinical data regarding 5 patients with nephrotic syndrome following ramucirumab treatment at our hospital from September 2016 to July 2018. All patient data regarding ramucirumab treatment were collected during review of electronic medical records. The following demographic and laboratory data were obtained for all participants: age, sex, proteinuria, hematuria, serum concentrations of creatinine (Cr) and albumin, estimated glomerular filtration rate (eGFR), and presence of comorbidities, including hypertension (HT), diabetes mellitus (DM), hyperlipidemia (DL), and chronic kidney disease (CKD).

Nephrotic syndrome was defined as both serum albumin less than 3 g/dL and urinary protein-to-creatinine ratio (UPCR) greater than 3.5. Proteinuria was defined as a urine dipstick test score ≥1+ or a urine protein creatinine ratio >0.3. Renal dysfunction was defined as an increase in serum Cr levels ≥50% or ≥0.3 mg/dL.^[10]^ Patients with renal dysfunction from an identifiable cause were not included. Patients with HT were defined as having systolic blood pressure ≥140 mmHg, diastolic blood pressure ≥90 mmHg, or received antihypertensive agents. Patients with DM were defined as those with a diagnosis of DM prior to baseline, those an HbA1c of 6.5% with a casual plasma glucose concentration of 200 mg/dL, or those receiving oral anti-diabetic agents or insulin. Patients with DL were defined as those with levels of total cholesterol ≥220 mg/dL, those with low-density lipoprotein cholesterol ≥140 mg/dL, triglyceride ≥150 mg/dL, or those receiving oral hypolipidemic agents. CKD was defined as an eGFR <60 mL/min/1.73 m^2^ for >3 months before the start of ramucirumab. Improvement of proteinuria and renal dysfunction were defined as normalization or decrease to baseline levels. Moreover, 2 renal biopsy specimens of metastatic colorectal cancer patients were evaluated by a pathologist.

### 2.2 Measurements

Blood and urine samples of all patients were collected for analysis upon admission to our hospital. Urine specimens were simultaneously measured using first-spot urine from the first urination of the morning. Urine Cr was measured using an enzymatic method, and total urine protein was measured using the turbidimetric method. The concentration of urinary total protein was normalized to 1 g/L urinary Cr and expressed as UPCR. Blood cell count and routine laboratory data were measured by the standard method using an automatic analyzer (Sysmex SF-3000; Hitachi, Tokyo, Japan). Serum Cr was measured using an enzymatic method (N-assay L-creatinine kit; Nittobo Medical Co., Tokyo, Japan). eGFR was calculated based on serum Cr concentration using the following formula: GFR (mL/min/1.73 m^2^) = 1.94 × Cr − 1.094 × Age − 0.287 ×  (0.739 if women), which was developed for Japanese patients by the Japanese Society of Nephrology due to inaccuracies in the Modification of Diet in Renal Disease equation for kidney disease in Asian populations.^[11]^

## 3 Results

### 3.1 Clinical and laboratory characteristics of patients before ramucirumab treatment

The demographic and laboratory characteristics of the patients before ramucirumab treatment are shown in Table 1. Patient age ranged from 50 to 84 years; 3 patients were men. There were 2 patients with gastric cancer and 3 with metastatic colorectal cancer. Three patients had HT, 3 had DL, 2 had CKD, and 1 had DM. Three patients had proteinuria, and none had hematuria. HT was uncontrolled only in Case 2. All patients had received prior chemotherapy before ramucirumab treatment.

**Table 1:**
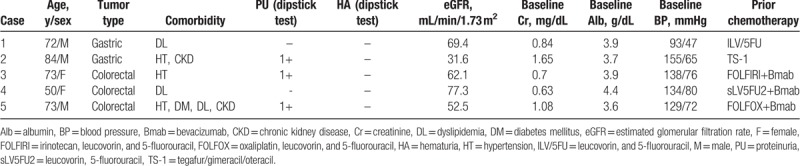
Demographics and laboratory characteristics of patients before ramucirumab treatment.

### 3.2 Clinical courses of patients following ramucirumab treatment

Table 2 shows the clinical courses of patients following ramucirumab treatment. All patients except Case 1 developed nephrotic syndrome after 1 or 2 doses of ramucirumab administration. The average time to clinical onset after ramucirumab treatment was 45.6 days (range 21–112 days). All patients had hypertension and hematuria. Two patients developed renal dysfunction. All patients received concomitant chemotherapy (2 patients received paclitaxel and 3 patients received irinotecan, leucovorin, and 5-fluorouracil). Severe hypoalbuminemia was only observed in Case 5.

**Table 2:**
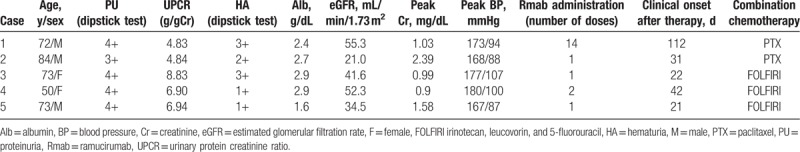
Clinical courses of patients after ramucirumab treatment.

### 3.3 Treatment and prognosis

The clinical and renal outcomes of patients with nephrotic syndrome after ramucirumab treatment are shown in Table 3. The mean follow-up period was 11.0 months (range 4–27 months). All patients with nephrotic syndrome discontinued ramucirumab treatment and started antihypertensive therapy with angiotensin receptor blockers, calcium blockers, and diuretics. Only Case 5 underwent one more round of ramucirumab administration before cessation of treatment. The average time to clinical improvement was 3.6 months (range 1–7 months). Two patients with renal dysfunction continued to exhibit proteinuria after drug discontinuation.

**Table 3:**
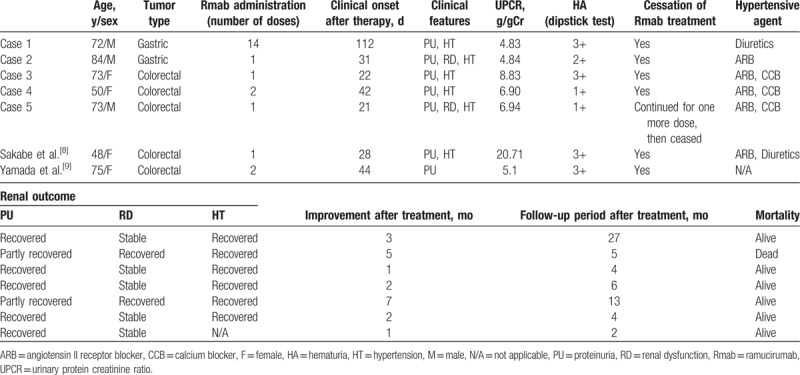
Treatment and renal outcome of patients undergoing ramucirumab treatment and review of literature.

### 3.4 Case 4 biopsy results

**Figure 1:**
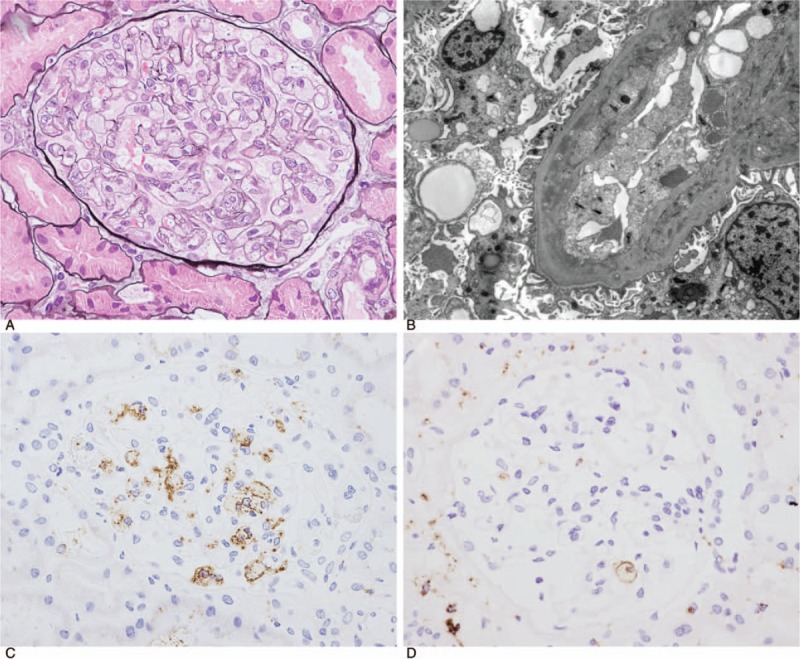
(A) Light microscopy revealed narrowing of the glomerular capillary lumina owing to diffuse glomerular basement membrane double contours (periodic acid-methenamine-silver [PAM] stain, ×400, high-power field, Case 4). (B) Electron microscopy revealed narrowed glomerular capillary lumina, double contours of the glomerular basement membrane, and partial foot process effacement (Case 5). (C, D) Immunoperoxidase staining for CD68. Glomerular infiltration of CD68-positive cells was observed (C, Case 4; D, Case 5).

Light microscopic examination revealed 14 glomeruli, 2 of which were globally sclerotic. The glomerulus showed diffuse double contours of the glomerular basement membrane (GBM), as well as widening of the subendothelial space (Fig. 1A). No fibrin thrombi were seen. Interstitial fibrosis, tubular atrophy, and intimal thickening of the arteries were mild. Immunofluorescence microscopy showed segmental fine granular glomerular capillary wall staining for IgA kappa and IgA lambda, but there was no IgG, IgM, or C3 staining. Electron microscopy revealed narrowed glomerular capillary lumina, double contours of the GBM, and an electron-dense deposit in the subendothelial space (Fig. 1B). Immunohistochemistry showed increased CD68 expression in the glomerular loop (Fig. 1C).

### 3.5 Case 5 biopsy results

Light microscopic examination revealed 17 glomeruli, 2 of which were globally sclerotic. The glomerulus showed double contours of the GBM. No fibrin thrombi were seen. Interstitial fibrosis and tubular atrophy were moderate. Moderate fibrosis and mild sclerosis were shown in arterial vessels. Immunofluorescence microscopy revealed negativity for IgG, IgA, IgM, and C3. Electron microscopy revealed subendothelial expansion and partial foot process effacement, whereas no electron dense depositions were detected. Immunohistochemistry showed increased CD68 expression in the glomerular loop (Fig. 1D).

## 4 Discussion

This study reports the clinicopathological features of nephrotic syndrome following ramucirumab treatment and revealed that nephrotic syndrome was a remarkably severe, rapidly developing adverse event following ramucirumab therapy. Renal biopsy revealed diffuse double contours, intracapillary foam cell infiltration, and partial foot process effacement. Ramucirumab-induced renal damage was reversible, and early drug discontinuation and antihypertensive therapy are recommended. To the best of our knowledge, this is the first case series to examine nephrotic syndrome associated with ramucirumab.

The mechanism by which ramucirumab results in renal dysfunction and leads to nephrotic syndrome is unclear. Anti-VEGF therapies are known to induce proteinuria with a mechanism attributable to inhibition of podocyte-derived VEGF, which maintains the regulation of the glomerular capillary wall and glomerular filtration barrier through VEGFR-2 expression on endothelial cells. Additionally, podocyte-derived VEGF signaling through VEGFR-2 is involved in the regulation of the glomerular filtration barrier.^[12]^ Furthermore, Sugimoto et al^[13]^ demonstrated that anti-VEGF treatment may cause direct damage to the podocytes with downregulation of nephrin. Thus, nephrotic syndrome following ramucirumab may be caused by direct damage to the podocytes with downregulation of nephrin, in addition to VEGFR-2 inhibition by ramucirumab. Moreover, proteinuria may also be caused by changes in blood flow dynamics, such as elevation of renal glomerular internal pressure due to HT, which may in turn block glomerular epithelial VEGF production.^[14]^ All of these factors may work in tandem in the development of nephrotic syndrome.

Nephrotic syndrome may rapidly develop after initiation of ramucirumab therapy. Nevertheless, there are few reports regarding the time of onset of high-grade proteinuria or nephrotic syndrome following therapy with anti-VEGF therapies. Sorich et al^[15]^ reported that the median time to high-grade proteinuria in metastatic renal cell carcinoma patients treated with pazopanib or sunitinib was 100 days. Table 3 shows the 5 cases in our study and previously reported cases of nephrotic syndrome following ramucirumab.^[8,9]^ Four of 5 patients in the present study and 2 previously reported cases developed nephrotic syndrome after 1 or 2 doses of ramucirumab. Urinalysis was not performed until 4 months after ramucirumab therapy in Case 1, and therefore the diagnosis of nephrotic syndrome was delayed. Since ramucirumab treatment may rapidly result in severe proteinuria, including nephrotic syndrome, compared with other anti-VEGF therapies, careful follow-up is recommended.

The 2 renal biopsies in the present study revealed GBM double contours, intracapillary foam cell infiltration, and partial foot process effacement. Both patients were diagnosed with renal limited thrombotic microangiopathy (TMA). Pathologically, renal injury during anti-VEGF therapy is composed of 2 different subtypes: glomerular diseases, including endothelial cell injury such as intraglomerular TMA, and podocytopathies, including minimal change disease (MCNS)/focal segmental glomerulonephritis (FGS).^[16]^ TMA lesions have been found more frequently following anti-VEGF therapy than following tyrosine kinase inhibitor therapy; in contrast, MCNS/FGS has been associated with both therapies, but is found less frequently in anti-VEGF antibody-treated patients.^[17,18]^ Pathological findings of ramucirumab-induced renal damage in this study were similar to those previously observed with anti-VEGF monoclonal antibody and VEGF trap treatment. As in the present study, Yamada et al^[9]^ reported TMA-like findings, endothelial damage, and GBM double contours in a renal biopsy from a patient with nephrotic syndrome following ramucirumab treatment. Pfister et al^[19]^ reported that, in patients with glomerular TMA with nephrotic-range proteinuria following treatment with anti-VEGF therapy, there were several characteristic findings, including GBM double contours, intracapillary foam cell infiltration, and partial foot process effacement. In addition, they hypothesized that inhibition of VEGF signaling between podocytes and endothelial cells resulted in endothelial injury that caused chronic glomerular TMA with GBM double contours with secondary evolution of foot process effacement. These findings may be characteristic of anti-VEGF therapy-induced TMA and are useful to differentiate nephrotic syndrome with renal TMA due to malignancy from drug-induced nephrotic syndrome with TMA. Moreover, as malignant tumors could cause various nephrotic syndromes (e.g., membrane nephropathy, MCNS, and FGS), renal biopsy should be performed.^[20–23]^

Ramucirumab-induced renal damage is reversible, and early drug discontinuation and antihypertensive therapy are recommended. In the present study and in 2 previously reported cases, patients with nephrotic syndrome recovered following drug discontinuation and administration of antihypertensive therapy. Previous reports showed rapid improvement of proteinuria, kidney dysfunction, and hypertension after discontinuation of anti-VEGF therapies.^[24,25]^ In the present study, 2 of 5 patients with ramucirumab-induced nephrotic syndrome maintained partial recovery of kidney function. Although drug effects are transient, nephrotoxicity is dose-dependent; therefore, early drug discontinuation is recommended after nephrotic syndrome diagnosis. Moreover, ramucirumab dosage should be adjusted based on proteinuria levels at baseline and during the course of treatment. When considering an effective antihypertensive therapy for ramucirumab-induced nephrotic syndrome, it may be required to consider the association of proteinuria induced by ramucirumab. Mechanisms underlying hypertension caused by anti-VEGF therapies include diminished nitric oxide synthesis in the endothelium, increased peripheral vascular resistance, and decreased density in the microvasculature, with additional contributions of renal dysfunction to hypertension.^[26]^ Among antihypertensive agents, angiotensin receptor blockers, angiotensin-converting enzyme inhibitors, and non-dihydropyridine calcium channel blockers may be preferred over other drugs.^[27]^ Diuretics are unlikely to be selected as a first-line agent due to diarrhea side effects of anti-VEGF therapies. Additionally, some calcium channel blockers should be avoided due to potential drug interactions. In addition, from the view of proteinuria, VEGF inhibition-induced proteinuria is associated with endothelial cell damage and slit membrane dysfunction, which are also caused by changes in blood flow dynamics, including elevation of renal glomerular internal pressure due to HT and decreased VEGF expression in glomerular epithelial cells.^[28]^ Considering these mechanisms, renin-angiotensin inhibitors and angiotensin-converting enzyme inhibitors may be effective for treating VEGF inhibition-induced hypertension and proteinuria.

There were several limitations in this study. First, the causes of proteinuria and renal dysfunction were not examined by pathology in all patients. Due to complication risks, renal biopsy is generally unfeasible in patients with cancer, especially those undergoing chemotherapy. We investigated only 2 cases of nephrotic syndrome by renal biopsy. Therefore, the results from this study can only be considered preliminary findings, and further confirmatory work is warranted before generating hypotheses. Second, it possible that the patients received other medications that may have led to nephrotic syndrome. However, the relationship between the onset of and recovery from nephrotic syndrome in concert with the initiation and cessation of ramucirumab treatment strongly suggests a link between ramucirumab and nephrotic syndrome in these patients. Finally, the follow-up duration was short. However, the main purpose of our study was to investigate the clinical courses and renal outcomes of patients who developed nephrotic syndrome after ramucirumab therapy. Most patients recovered from nephrotic syndrome and other renal adverse events, including renal dysfunction and hypertension.

In conclusion, severe renal adverse events, especially nephrotic syndrome, occurred after ramucirumab therapy. Based on the present results, we suggest close monitoring of urinalysis, renal function, and blood pressure in patients receiving ramucirumab. When severe renal adverse events, such as nephrotic syndrome, are observed, early drug discontinuation and antihypertensive therapy are recommended. Future studies focusing on the analysis of risk and lifestyle factors that might explain ramucirumab induced nephrotic syndrome are required.

## Acknowledgments

The authors deeply thank Dr. Keiji Takahashi in the Department of Chemotherapy, Tokyo Metropolitan Komagome Hospital, for his invaluable help in conducting the study.

## Author contributions

**Investigation:** Kentaro Kawasoe.

**Project administration:** Akihito Ohta.

**Supervision:** Kosaku Nitta.

**Validation:** Akiko Tonooka.

**Writing – original draft:** Teruhiro Fujii.
